# Frustration reactions as a factor of adherence to treatment in patients with cardiovascular diseases

**DOI:** 10.1192/j.eurpsy.2025.1227

**Published:** 2025-08-26

**Authors:** E. V. Deshchenko, E. I. Pervichko

**Affiliations:** 1 Lomonosov Moscow State University, Moscow, Russian Federation

## Abstract

**Introduction:**

Adherence to treatment plays a key role in the effectiveness of therapy of the cardiovascular diseases. Patient’s personality becomes an inevitable determinant of health behaviour influencing the way the patient reacts to the illness itself. The painful sensations, the need to adapt to the disease may be the cause of frustration in patients (Heszen-Klemens. Soc Sci Med 1987, 24 (5) 409-416). Hence, it is necessary to assess how the frustration reactions are connected with the adherence to treatment.

**Objectives:**

The aim of the research was to study the relationship between the frustration reactions and the adherence to treatment in patients with cardiovascular diseases.

**Methods:**

The Picture Frustration Test (Rosenzweig. Journal of Personality 1945, 14 3-23) was used to assess frustration reactions of the patients. The Questionnaire for Comprehensive Assessment of Treatment Adherence was used to provide a complex evaluation of the adherence to treatment (Nikolayev, Skirdenko. Clinical Pharmacology and Therapy 2018, 1 74-78). The study was conducted from January 2024 to April 2024. The sample consisted of 42 male patients hospitalised with multiple cardiac pathology, whose average age was 49.40±7.71.

**Results:**

The average adherence to treatment of the patients with cardiovascular diseases in our sample was 61.17±18.53%, with twelve (30%) participants being defined as low-adherent and nine (22.5%) as high-adherent. What concerns direction of frustration reactions, low-adherent patients were more likely to exhibit extrapunitive reactions (H=7,760, p=0,021), whereas high-adherent patients demonstrated intropunitive reactions more often (H=6,062, p=0,048). More interestingly, there were significant differences in types of frustration reactions, with need-persistent reactions being more characteristic for the high adherent-patients (H=6,551, p=0,038). Intropunitive and need-persistent frustration reactions were associated positively with the adherence to treatment (r=0.428, p=0.013; r=0.459, p=0.007). Extrapunitive reactions were found to be negatively associated with the adherence (r=-0.409, p=0.004).

**Conclusions:**

Our study was the first to consider the connection between the frustration reactions of the patients with cardiovascular diseases and their adherence to treatment. The results indicate that the way in which patients typically react to the frustration is connected with the way in which they handle limitations and requirements of the treatment process. When the patient is more likely to react to frustration in problem-solving manner, the chances are that their health behaviour will also lead to a sufficient adherence to treatment.

**Disclosure of Interest:**

None Declared

